# Dengue-Induced Hemophagocytic Lymphohistiocytosis: A Case Report and Literature Review

**DOI:** 10.7759/cureus.20172

**Published:** 2021-12-05

**Authors:** Adeeb Munshi, Anas Alsuraihi, Marwan Balubaid, Mohammad Althobaiti, Abdulhakeem Althaqafi

**Affiliations:** 1 Infectious Diseases, King Abdullah International Medical Research Center/King Saud bin Abdulaziz University for Health Sciences College of Medicine, Ministry of National Guard-Health Affairs, Jeddah, SAU; 2 Adult Hematology, King Abdullah International Medical Research Center/King Saud bin Abdulaziz University for Health Sciences College of Medicine, Ministry of National Guard-Health Affairs, Jeddah, SAU; 3 Internal Medicine, King Abdullah International Medical Research Center/King Saud bin Abdulaziz University for Health Sciences College of Medicine, Ministry of National Guard-Health Affairs, Jeddah, SAU; 4 Infectious Diseases, King Abdulaziz Medical City, King Saud Bin Abdulaziz University for Health Sciences, King Abdullah International Medical Research Center, Jeddah, SAU

**Keywords:** cytopenia, hepatomegaly, hyperferritinemia, dengue fever, hemophagocytic lymphohistiocytosis

## Abstract

Hemophagocytic lymphohistiocytosis (HLH) is an uncommon fatal disease of otherwise normal but hyperactive lymphocytes and histocytes. HLH could be primary (hereditary) or secondary (acquired). Fever, hepatosplenomegaly, lymphadenopathy, and neurologic dysfunction are among the common symptoms of HLH. The diagnosis of HLH is based on clinical and biochemical findings. We report here a case of a patient infected with the dengue virus who developed HLH during hospitalization. A 63-year-old female known case of asthma on inhalers, chronic hepatitis B virus, gastritis on proton pump inhibitors, and hemoglobin H disease presented to the emergency department (ED) with a history of high-grade fever (highest recorded temperature 40° C/ 104° F), which was relieved partially by antipyretics, generalized fatigability, body aches, headache and mosquito bites for four days. The physical examination was significant for hepatomegaly of 4 cm below the right costal margin. Investigations revealed pancytopenia with elevated ferritin levels (> 40000 µg/L). Viral serology was positive for dengue NS1 antigen. After hematology consultation, a bone marrow biopsy was done, which showed trilineage hematopoiesis with increased histiocytes and occasional hemophagocytosis. Given that the patient was clinically stable and there was a clear triggering condition, we opted for supportive measures rather than HLH-specific therapy. The patient was given 2 units packed red blood cells for anemia. On the following days, the patient has no recurrence of fever, with marked improvement in the biochemical profile including ferritin level (1165 µg/L). HLH is a deleterious disease with a high fatality rate, which requires the clinician to have a low threshold for suspicion in the differentials of children and adults with symptoms of persistent fever, hepatosplenomegaly, and cytopenia. Dengue-associated HLH diagnosis is challenging, but it is very important to be recognized, as early recognition is associated with better outcomes. Physicians must work in collaboration with pathologists and microbiologists for the proper diagnosis.

## Introduction

Hemophagocytic lymphohistiocytosis (HLH) is an uncommon syndrome. Jaundice, splenomegaly, fever, and the key feature of phagocytosis by macrophages, leukocytes, platelets, erythrocytes, and their precursors in the bone marrow and other tissues characterize it. This phenomenon is known as hemophagocytosis [1].

HLH is primarily caused by mutations in genes that are responsible for the production of cytotoxic T cells and natural killer (NK) cells. These are used to kill cells infected with pathogens like the Epstein-Barr virus (EBV) or the dengue virus [2]. The following genes are included in these mutations: STX11, SH2D1A, PRF1, UNC13D, and ITK [3-4].

Secondarily, it can be triggered by malignant or non-malignant diseases, which have a tendency to damage the immune system. Malignant disorders include acute lymphocytic leukemia, acute myeloid leukemia, B-cell lymphoma, T-cell lymphoma, and myelodysplastic syndrome. Non-malignant disorders linked to HLH are juvenile idiopathic arthritis, juvenile Kawasaki disease, systemic lupus erythematosus (SLE), Still’s disease - juvenile and adult-onset, and rheumatoid arthritis (RA) [5].

Infection caused by EBV, human immune deficiency virus (HIV), dengue virus, cytomegalovirus (CMV), bacteria, fungi, protozoa, and now possibly severe acute respiratory syndrome coronavirus 2 (SARS-COV-2) can be a cause of secondary HLH [6]. Iatrogenic causes like organ transplantation, chemotherapy, or immunosuppressive therapy can also result in secondary HLH [7].

Whether is it is an inherited HLH or an acquired one, it shares common pathophysiology. In a simplified way, it is suggested that it leads to an unchecked immune response while encountering the triggers. The hallmark of HLH is NK cell cytotoxicity. Familial HLH is linked to granule-dependent cytotoxicity. The inability to eliminate antigen-presenting cells and infected ones leads to uncontrolled proliferation and activation of the immune response with abundant cytokines. The clinical picture of HLH is due to the invasion of these cells in the organs and releasing more cytokines. Interleukin 1 (IL-1), IL-6, and tumor necrosis factor (TNF)-alpha cause the fever. The suppression of hematopoiesis by TNF-alpha and TNF-gamma results in cytopenia. Activated macrophages release ferritin and plasminogen activator, which leads to hyperfibrinolysis [8].

HLH is a syndromic disease, characterized by a pattern of the clinical picture. Every individual may show a range of signs caused by pathological inflammation. Along with the clinical signs, genetic testing remains the most desired criteria for the confirmation of the disease. Moreover, genetic testing helps in the prediction of the prognosis and recurrence of the disease, as well as the determination of the predisposition in the asymptomatic siblings. The Histiocyte Society proposed the standard for the diagnosis in 1994. This was revised in 2004 and is considered a definition of the criteria for the diagnosis of HLH [9].

According to this criterion, the diagnosis of HLH can be established either on the confirmation of the molecular diagnosis, e.g., pathological mutations of PRF1, UNC13D, Munc18-2, Rab27a, STX11, SH2D1A, or BIRC4, or on five or more of the following clinical findings: fever ≥ 38.5°C, splenomegaly, cytopenia, hypertriglyceridemia, hemophagocytosis in bone marrow, spleen, lymph nodes, or liver, low or absent NK cell activity, elevated CD25 (α-chain of SIL-2 receptor), and ferritin> 500 µg/L [10].

Due to its rarity, range of presentation, and non-specific findings, the diagnosis of HLH becomes very challenging. Practical considerations include the proper assessment and detection of the signs of HLH, mainly in critically ill patients. CD25 is a useful inflammatory marker in the workup for HLH. Another valuable marker is ferritin level, which if more than 10,000 µg/L, becomes a very sensitive and specific indicator for the diagnosis of the HLH [11].

## Case presentation

A 63-year-old female, known case of asthma on inhalers, chronic hepatitis B virus, gastritis on proton pump inhibitors, hemoglobin H disease (which was confirmed by molecular test), presented to the emergency department (ED) with a history of high-grade fever (highest recorded temperature 40° C/ 104° F), which was relieved partially by antipyretics, generalized fatigability, body aches, and headache for four days. It was associated with dry cough, diffuse abdominal pain, and multiple bouts of non-bloody vomiting and diarrhea. The patient had a history of mosquito bites. Besides, the patient complained of two episodes of red urine at the time she arrived at the ED. The patient denied a history of shortness of breath (SOB), chest pain, change in weight or appetite, or contact with a sick patient. The further systemic review was unremarkable. On examination in the ED, the blood pressure was 110/53 mm Hg, heart rate was 78 beats per minute, respiratory rate was 20 breaths per minute, and oxygen saturation was 99% while on ambient air. The cardiopulmonary examination was normal. The abdominal examination showed hepatomegaly of 4 cm below the right costal margin. The remainder of the examination was unremarkable.

Laboratory investigations revealed leukopenia (WBC: 2.1x109/L), anemia (Hg: 7.9 g/dL), with low mean corpuscular volume (MCV) and mean corpuscular hemoglobin (MCH), mild thrombocytopenia (platelets: 122x103/ µL). The iron study showed a high ferritin level (> 40000 µg/L), an iron of 10.0 µmol/L, transferrin of 1.60 g/L, and transferrin saturation of 73%. Kidney and liver functions were normal (Table 1). Urinalysis showed a moderate amount of blood in the urine. The blood, urine, stool cultures were negative. The coronavirus disease (COVID) nasopharyngeal swab was negative as well. Chest X-ray showed no airspace opacity, effusion, or pneumothorax. Computed tomography of the abdomen was not remarkable. The first impression was query viral illness, and the patient was treated supportively with intravenous fluid and antipyretics.

**Table 1:** Investigations of the patient MCV: mean corpuscular volume; INR: international normalized ratio; HIV: human immunodeficiency virus; ALT: alanine aminotransferase; AST: aspartate aminotransferase; LDH: lactate dehydrogenase

Variable	Upon presentation
Haemoglobin (11.5 ~ 16.5 g/dL)	7.9
Hematocrit (40 - 54%)	24.4
White cell count (4 - 11x10^9^/L)	2.1
Lymphocyte (1.5 - 4x10^9^/L)	0.97
Neutrophils (2- 7.5x10^9^/L)	0.97
MCV (76 ~ 96 fL)	55
Platelet (150 - 450 x10^9^/L)	122
INR (0.8 – 1.2)	1.2
Creatinine (50 – 74 µmol/L)	67
AST (5 – 34 IU/L)	138
ALT (6 – 28 U/L)	62
Total Bilirubin (2.1 – 15.5 µmol/L)	24.7
Direct Bilirubin (0 – 9 µmol/L)	9
Ferritin (24 – 336 µg/L)	>40000.00
Transferrin (2.03 – 3.96 g/L)	1.60
Transferrin Saturation (%)	73%
Dengue NS1 Antigen	Positive
Dengue IgG	Negative
Dengue IgM	Negative
Coronavirus (Qualitative)	Not Detected
HIV	Negative
Malaria Smear	Negative
LDH (100 – 217 U/L)	863
Haptoglobin (.360 – 1.950 g/L)	<0.058
Fibrinogen (2 – 4 g/L)	1.89
D-Dimer (0 – 0.50 mg/L)	1.37
Cholesterol Total (~ 5.18 mmol/L)	2.72
Triglyceride (< 1.70 mmol/L)	1.36

The hemolysis workup was sent (lactate dehydrogenase (LDH), reticulocyte count, indirect bilirubin, and platelets); a viral panel, including dengue and malaria smear, was also ordered (Table 1). Blood smear showed leucopenia with an increase in lymphocytes, microcytic hypochromic, polychromasia, teardrops, elliptocytes, target cells, and mild thrombocytopenia. The hematology team was consulted, and their impression was HLH, for which a bone marrow biopsy was conducted.

**Figure 1:**
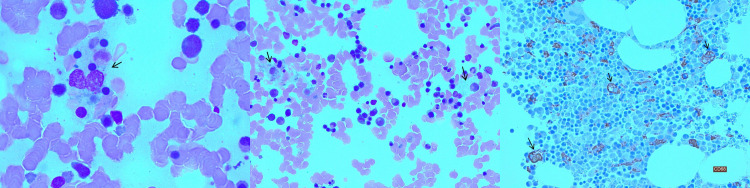
Bone marrow aspirate and biopsy showing increased cellularity for age at 55%, trilineage hematopoiesis with increased histiocytes, and occasional hemophagocytosis (arrows)

Bone marrow aspirate and biopsy showing trilineage hematopoiesis with increased histiocytes and occasional hemophagocytosis (Figure 1). Given that the patient was clinically stable and there was a clear triggering condition, we opted for supportive measures rather than HLH-specific therapy. The patient was given 2 units packed red blood cells for anemia. On the following days, the patient has no recurrence of fever, with marked improvement in the biochemical profile, WBC was 6.3x109/L, Hg was 9 g/dL, platelet was 238x109/µL, and ferritin level was 1165 µg/L.

## Discussion

Hemophagocytic lymphohistiocytosis is an aggressive and rare disease. The most affected population is infants, from birth until 18 months of age. However, children and adults can be affected as well. The presentation of HLH initially can resemble the presentation of other common infections. Fever and multi-system involvement is the hallmark of the disease [1].

The pathophysiology behind HLH is not well-understood. One proposed hypothesis is that an increased number of activated macrophages as a result of improper proliferation and activation of T-cells. These activated macrophages have lost their ability to kill intracellular macrophages. Perforin and NK cells are implicated in the HLH subtypes. In the case of perforin deficiency, the defensive mechanisms against intracellular pathogens are affected. While in the case of decreased NK cell activity, T-cells will be more activated and this will lead to the production of large numbers of cytokines such as IFN-g, TNF-a, and GM-CSF. The previous process will lead to persistent macrophage activation [12].

Fever, splenomegaly, cytopenia, hypertriglyceridemia, hypofibrinogenemia, and hemophagocytosis used to be enough for the diagnosis of HLH. These criteria were modified in 2004 and the Histiocyte Society added three more criteria: low or absent NK cell activity, hyperferritinemia, and high-soluble interleukin-2-receptor levels. The diagnosis should include five out of the previous eight criteria [10-13].

HLH can be divided into two types. The first type is primary HLH, which means there is a background of genetic disease. The second type is secondary HLH, which means it is attributed to infectious, malignant, rheumatologic, or other secondary causes. However, the infection can be as well a trigger for primary HLH [13].

The majority of secondary HLH cases are due to infectious causes, infection-associated hemophagocytic syndrome (IAHS). EBV is the most common cause. CMV, parvovirus, herpes simplex virus (HSV), varicella-zoster virus (VZV), measles virus, human herpesvirus 8, H1N1 influenza virus, parechovirus, and HIV all are also implicated in the pathogenesis of secondary HLH [10-12-14].

Dengue-associated HLH has been well-reported in children, however, only a few case reports have been identified in adults. Dengue is the most common arthropod-borne viral illness in humans. The clinical manifestations of dengue can range from asymptomatic disease to dengue hemorrhagic fever (DHF) and dengue shock syndrome (DSS). Four dengue viruses (DENV) have been identified as causative agents. These are DENV1, DENV2, DENV3, and DENV4. Out of the previous four dengue viruses, DENV1, DENV3, and DENV4 have been identified to cause HLH. Due to the increasing number of dengue detection every year, dengue-associated HLH has increased as well [13-15].

A study investigated dengue-associated HLH cases and described that infants were more affected and cases were related to higher morbidity (100% ICU admission and longer stay) with a mortality of 4.5% more than dengue patients. As expected in the cases of HLH, dengue-associated HLH also developed anemia, splenomegaly, hepatomegaly, along with elevated aminotransferases. Contrarily, neutropenia and thrombocytopenia weren’t observed in dengue-associated HLH patients. This feature can be due to the clinical characteristics of dengue. It was also observed that morbidity was higher in dengue-related HLH as compared to dengue alone [16].

Another study conducted on 180 dengue patients concluded that high dengue-HLH mortality makes it a candidate for a short course of HLH-directed treatment in specific patients. Prednisolone has been linked with lesser derangement in leukocyte and AST levels [17]. Treatment of HLH with dexamethasone and etoposide has shown a significant reduction in mortality in EBV-infection-related HLH [17-19].

T-cells are infected in EBV-HLH and dengue-induced-HLH. Additionally, in dengue, T-cells play a part in the viral replication and secretion of viral particles. So the treatment of dengue-induced HLH by the same regime as EBV-HLH provides a rationale as the corticosteroids and etoposide reduce the lymphocytes [17-20].

In the treatment of primary and secondary HLH, etoposide has proven to be instrumental [21-22]. In a study of 162 patients, etoposide was used as the first-line treatment showing better outcomes (P=.079) [23]. 

In a study from Puerto Rico, which studied dengue-HLH in children, etoposide again showed better results [16]. The exact mechanism of etoposide in hyper inflammation isn’t well understood, but its involvement in the selective deletion of activated T-cells and reduction of inflammatory cytokines improves the conditions of HLH [24].

Treatment of HLH with dexamethasone is also considered very beneficial. Eight out of 10 patients with severe disease survived after the administration of dexamethasone in a study [16]. Literature is suggestive that the use of steroids in the HLH in severe dengue patients requires more study [16,25-26]. It is observed that the levels of ALT, AST, creatinine, LDH, and ferritin remain significantly higher in severely affected patients. Increasing AST and peak ferritin levels are linked with higher fatality. Physicians must consider HLH in dengue-infected patients if they observe persistent fever, abnormal mental state, cytopenia with organ issue, and, importantly, ferritin greater than 10,000 μg/L [26].

Dengue-associated HLH can be treated by targeting the underlying cause as in our patient. However, due to its anti-inflammatory effect, pulse dose glucocorticoids (methylprednisolone or dexamethasone) can be used in the treatment of dengue-associated HLH. Intravenous immunoglobulin G can be used either alone or with dexamethasone or methylprednisolone. Surprisingly, anakinra, an interleukin-1 inhibitor, has been reported by Sakshi Bami et al. to have promising outcomes in treating secondary HLH (with or without dexamethasone), with the advantage of deliberately avoiding etoposide [26-27]. The dengue-associated HLH diagnosis is challenging but it is very important to be recognized, as it is associated with better treatment options [26-27].

## Conclusions

HLH should be considered in the differential diagnosis of children and adults with symptoms of persistent fever, hepatosplenomegaly, and cytopenia. Early intervention and recognition of the disease with the direction of appropriate therapy may significantly improve the patient’s condition. Physicians must work in collaboration with pathologists and microbiologists for the proper diagnosis. Clinicians must be aware that HLH can occur in dengue patients, especially in the dengue-prevalent regions of the world.
